# Feasibility and acceptability of video‐based microinterventions for eating disorder prevention among adolescents in secondary schools

**DOI:** 10.1002/eat.23781

**Published:** 2022-07-19

**Authors:** Mhairi Kristoffersen, Catherine Johnson, Melissa J. Atkinson

**Affiliations:** ^1^ Department of Psychology University of Bath Bath UK; ^2^ College of Education, Psychology, and Social Work Flinders University Adelaide South Australia Australia

**Keywords:** adolescent, body image, cognitive dissonance, feeding and eating disorders, media pressures, microintervention, risk factors, schools, self‐compassion

## Abstract

**Objective:**

Eating disorders (EDs) often emerge in late adolescence. Schools are ideal settings for prevention programs; however, cost and time limit implementation. Microinterventions may overcome these challenges. This study adapted two microinterventions (cognitive dissonance, self‐compassion) and assessed feasibility and acceptability among mid‐adolescents to provide proof‐of‐concept for further investigation.

**Method:**

Feedback from staff (*n* = 5) and student (*n* = 15) focus groups contributed iteratively to the adaptation of intervention materials. Students in Grade 10 and 11 (*N* = 101, *M*_age_ = 15.80, *SD* = 0.68) were then randomly allocated by class to a 20‐min video‐based cognitive‐dissonance or self‐compassion intervention, accessed on their school devices. ED risk and protective factors were assessed at baseline, immediate postintervention (state outcomes), and 1‐week follow‐up (trait outcomes). Acceptability items were included at both timepoints.

**Results:**

Implementation was deemed feasible. Girls generally reported greater acceptability than boys. Among girls, the self‐compassion intervention demonstrated greater acceptability. Among boys, some aspects of acceptability (e.g., lesson endorsement, utilization of techniques) were rated higher in the cognitive dissonance group whereas other aspects (e.g., understanding, interest) were greater in the self‐compassion group. All groups exhibited favorable changes in most state outcomes, however trait outcome change was varied.

**Discussion:**

Microinterventions provide a feasible way of implementing prevention strategies in a time‐poor educational context. Future large‐scale evaluation is warranted to determine efficacy, following modifications based on current findings.

**Public Significance:**

This study shows promising feasibility and acceptability of two brief, self‐guided video‐based lessons (microinterventions) for adolescents in school classrooms, that use psychological techniques to target appearance pressures as a key risk factor for eating disorders. Such interventions are easier to implement in school settings than longer, facilitator‐led interventions, to encourage greater uptake and ongoing use. Findings support further research to evaluate effectiveness, to ultimately provide accessible and gender‐inclusive tools for busy schools.

## 1 INTRODUCTION

Eating disorders (EDs) are complex disorders characterized by significant physical and psychological impairment (Herpertz‐Dahlmann, 2009), high mortality and comorbidity rates (Smink et al., 2012), and a chronic and relapsing illness course (Fernández‐Aranda et al., 2021). Typically, EDs emerge in late adolescence (Allen et al., 2013); however, ED risk factors present earlier, emphasizing the need for prevention earlier in adolescence (Lacroix et al., 2022; Stice & Van Ryzin, 2019).

There are several empirically supported risk factors in the development of ED which provide crucial intervention targets, including appearance pressures and internalization of sociocultural ideals, weight and shape concerns, and negative affect (Pennesi & Wade, 2016; Stice, Gau, Rohde, & Shaw, 2017). More recently, attention has included potential ED protective factors such as body appreciation, defined as appreciating the features, functionality and health of one's body irrespective of congruence with sociocultural ideals (Tylka & Wood‐Barcalow, 2015). Targeting protective factors may be especially useful when delivering and evaluating programs to low‐risk individuals—as is often the case for preventive interventions—by building resilience against future pressures (Levine & Smolak, 2015). Additionally, given the widespread promotion of both thin and muscular sociocultural ideals presented in the media (Karazsia, Murnen, & Tylka, 2017), and the daily exposure to and use of media among young people (Kelly, Zilanawala, Booker, & Sacker, 2018), focusing interventions on navigating appearance pressures is highly important and relevant for adolescents.

ED prevention has often focused on high‐risk females. The increasing prevalence of body dissatisfaction, among other ED risk factors, in adolescent boys (Murray et al., 2018), and the high prevalence of ED risk factors in gender non‐binary and transgender individuals (Gordon et al., 2021) indicate a need for prevention efforts to target all adolescents, regardless of gender identity. Not only is a universal approach a cost effective and feasible way to disseminate inclusive ED prevention programs, but also understanding the perspective of others is likely to be beneficial for those who feel less affected by the topic (Dunstan et al., 2017).

Schools are ideal settings for the implementation of universal prevention programs owing to a broad audience primed for learning (Yager et al., 2013). Nevertheless, current programs are often not practical for schools, requiring multiple, lengthy sessions with a trained provider (Arnold et al., 2021). Microinterventions are a promising alternative. They are short, self‐guided, and target specific symptoms/risk factors to provide focused benefit with either single or repeated use (Elefant et al., 2017). Microinterventions have been developed to target ED risk and protective factors in adults and early adolescents with promising results (e.g., Fuller‐Tyszkiewicz et al., 2019; Matheson et al., 2020). However, microinterventions have not been assessed within a school context, and may prove useful among senior students where academic curricula are tightly packed and coincide with a time of salient body image concerns (Bucchianeri et al., 2013).

A recent study by Atkinson and Diedrichs (2021) evaluated two video‐based microinterventions for responding to appearance‐ideal media as a key influence in the development of EDs, using cognitive‐dissonance and mindfulness techniques, respectively. They found support for both interventions in providing immediate and short‐term (1‐week) benefits to key ED risk (e.g., appearance‐ideal internalization) and protective (e.g., body appreciation) factors in a sample of 202 young adult women. The current study aimed to adapt these microinterventions for school‐based delivery to mixed gender mid‐adolescents. However, mindfulness‐based programs have had varied impact among adolescents, which may partly result from poor comprehension of abstract concepts (Johnson et al., 2017). As the brevity of microinterventions confers minimal time to grasp concepts, we opted to focus the second intervention on self‐compassion as a related concept that is potentially more accessible and has shown benefit in an alternative intervention format with adolescents (Rodgers et al., 2018). Our specific aims were therefore to:

1. Adapt the microinterventions developed by Atkinson and Diedrichs (2021) through student and teacher consultation.
2. Determine feasibility of implementing interventions in secondary schools, and research methods necessary for further large‐scale evaluation.
3. Assess acceptability among students.
4. Explore within‐group changes in state and trait outcomes, by gender, to indicate direction of any change, detect potential for harmful iatrogenic effects, and inform feasibility of assessment methods.

## 2 METHOD

### 2.1 Design

This study used a mixed‐methods, Person‐Based Approach (PBA; Yardley et al., 2015) focusing on accommodating the perspective of the individuals who will use the intervention. PBA consists of three phases: design, development, and implementation.

During the design phase, a literature review identified gaps in current ED prevention literature and helped to inform intervention selection and the guiding principles for initial adaptation. Four main objectives were decided on as the guiding principles:

1. To improve accessibility and feasibility of interventions targeting ED risk and protective factors for mid‐adolescents. To meet this objective the interventions needed to provide content through widely accessible means, for example be delivered in schools or online, not require a facilitator, and be time limited.
2. To make the intervention accessible and relevant to all genders. To meet this objective, it was essential the intervention provide content that was not exclusive to one gender, use gender inclusive language and provide content that males, non‐binary and transgender individuals could relate to as this is often missing in current interventions.
3. To provide techniques that are easy to understand and utilize in day‐to‐day life. Thus, the intervention needed to provide comprehensive educational content about techniques adolescents could use to readily respond to media body ideals, as well as provide easy exercises to help promote understanding and provide opportunities for further practice and consolidation (e.g., take‐home sheets).
4. To ensure content is acceptable to mid‐adolescents. The intervention should utilize examples, language and terminology that is understood and relatable to mid‐adolescents.

The development phase involved consultation with school staff, via written feedback, and students, through focus groups. Analysis was iterative, with modifications to the intervention occurring after each consultation. The implementation phase comprised a randomized feasibility study.

### 2.2 Participants and procedure

**FIGURE 1:**
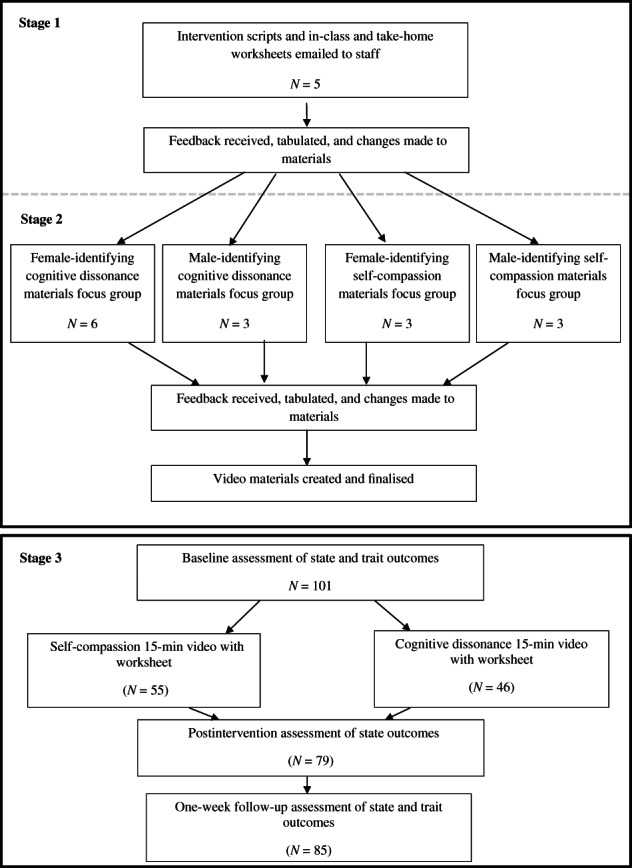
CONSORT flow diagram of participation throughout stages 1, 2, and 3.

The study took place at a private, coeducational secondary school in South Australia. For the development phase, school staff (*N* = 5) were emailed the adapted intervention materials (see Figure 1). They were asked to record any comments as tracked changes within the documents and as general comments within their return emails. Staff were asked to specifically comment on the age appropriateness of the materials and feasibility of implementation. Only Grade 10 students were invited to participate in the student focus groups due to exams taking place for Grade 11 students. *N* = 15 students provided feedback within four focus groups, with materials presented to separate groups of self‐identifying girls and boys for self‐compassion and cognitive‐dissonance, respectively.

For the implementation phase, Grade 10 and 11 students were invited to participate (see Figure 1). Those who participated in the focus groups were invited to participate again in this phase. Active (opt‐in) informed consent was obtained from parents and assent from students prior to baseline. *N* = 101 students aged 15 to 17 (*M* = 15.80, *SD* = 0.68) participated. Within each grade, classes were randomly assigned to the cognitive‐dissonance intervention (27 girls, 18 boys, 1 non‐binary) or self‐compassion intervention (27 girls and 28 boys).

The study was designed for students to engage with the microintervention video individually on electronic devices with an accompanying individual paper worksheet, under teacher supervision. Due to technical issues, Grade 10 students viewed the intervention on the classroom screen. All students completed a postintervention survey online and received a take‐home sheet. One‐week later, students completed a follow‐up online survey. This follow up period is in line with current microintervention research where follow up periods span 1–3 weeks (Atkinson & Diedrichs, 2021; Fuller‐Tyszkiewicz et al., 2019; Gobin, McComb, Mills, 2022; Matheson et al., 2020).

The interventions were designed to be “plug and play”, with no teacher training or direct involvement indicated. Class teachers were sent an email 1‐week prior to data collection containing instructions for the lesson, including links to the surveys and videos. Furthermore, due to the personal nature of the activities on the take‐home sheet and feedback from the focus groups, the sheets were not presented as compulsory. Instead, the benefit of practice was highlighted in the video, on the sheet and by the classroom teachers.

## 3 MATERIALS

### 3.1 Interventions

Interventions were adapted from Atkinson and Diedrichs (2021) by the authors. They consisted of a 20‐min video with two young adult presenters (one female, one male) accompanied by in‐class and take‐home worksheets. The cognitive‐dissonance intervention focused on the costs of pursuing media appearance ideals and practice challenging these. The self‐compassion intervention focused on the costs of being self‐critical and practice being self‐compassionate (see Table S1 for further detail).

### 3.2 Measures

An outline of measures assessing state and trait outcomes, and acceptability items, is displayed in Table 1.

**TABLE 1:** Description and reliability of measures assessing state, trait, and acceptability outcomes

Variable	Measure name	Measure information	(*α*)	(*α*)
*State*			Girls	Boys
Risk factors	Visual Analogue Scales (VAS; Heinberg & Thompson, 1995).	8 separate scales measuring weight satisfaction, weight distress, appearance satisfaction, appearance distress, appearance‐ideal internalization, pressures, pressure distress, and positive mood in the here and now. Rated on electronic sliders from 0 (*not at all*) to 100 (*very much*) (Atkinson & Diedrichs, 2021)	—	—
*Trait*				
Weight and shape concerns	Eating Disorders Examination Questionnaire (EDE‐Q; Fairburn & Beglin, 1994)—Weight and Shape Concerns Subscale	12 items (e.g., *“how dissatisfied have you felt about your shape”*) scored on a 7‐point Likert‐Scale from 0 (*Not at all*) to 6 (*markedly*). A high score is indicative of greater weight and shape concerns.	.97	.93
Body appreciation	Body Appreciation Scale 2‐Children (BAS‐2C; Halliwell, Jarman, Tylka & Slater, 2017)	10 items (e.g., *“I feel love for my body”*) scored on a 5‐point Likert Scale from 1 (*never*) to 5 (*always*). Higher scores indicate greater body appreciation.	.96	.93
Appearance ideal internalization	Sociocultural Attitudes toward Appearance Scale‐ 4 (SATAQ‐4; Schaefer et al., 2015) Internalization Thin/Low Body Fat subscale	5 items (e.g., *“I want my body to look very thin”*) scored on a 5‐point Likert scale from 1 (Definitely Disagree) to 5 (Definitely Agree). Higher scores indicate greater thin‐ideal internalization.	.87	.80
Athletic ideal internalization	Sociocultural Attitudes toward Appearance Scale‐ 4 (SATAQ‐4; Schaefer et al., 2015) Internalization Muscular/athletic subscale	5 items *(*e.g., *“I think a lot about looking muscular”)* scored on a 5‐point Likert scale from 1 (Definitely Disagree) to 5 (Definitely Agree). Higher scores indicate greater muscular/athletic ideal internalization.	.92	.92
Perceived sociocultural pressures	Sociocultural Attitudes toward Appearance Scale‐ 4 (SATAQ‐4; Schaefer et al., 2015) Pressures—Media subscale	4 items (e.g., “*I feel pressure from the media to look in better shape”)* scored on a 5‐point Likert scale from 1 (Definitely Disagree) to 5 (Definitely Agree). Higher scores indicate greater perceived pressure from the media.	.93	.95
Positive and negative affect	Positive and Negative Affect Schedule for Children (PANAS‐C; Ebesutani et al., 2012)	5 positive affect items (PA; e.g., Cheerful) and 5 negative affect items (NA; e.g., Miserable) examining extent of feelings over the past week from 1 (*not at all*) to 5 (*extremely*). Higher scores on positive items indicate greater positive affect, and higher scores on negative items indicate greater negative affect.	.92 (PA) .89 (NA)	.87 (PA) .85 (NA
Self‐compassion	Self‐Compassion Scale Short form (SCS‐SF; Raes et al., 2010)	12 items (e.g., *“I'm disapproving and judgmental about my own flaws and inadequacies”*) scored on a 5‐point Likert scale ranging from 1 (almost never) to 5 (almost always). High scores are indicative of greater self‐compassion.	.88	.75
Self‐criticism	Forms of Self‐criticizing/attacking and Self‐reassuring Scale (FSCRS‐SF; Sommers‐Spijkerman et al., 2018)	14 items (e.g., *“I find it easy to forgive myself”*) split into three subscales, inadequate self (IS; 5 items), hated self (HS; 4 items) and reassured self (RS; 5 items) scored on a 5‐point Likert scale from 0 (not at all like me) to 4 (extremely like me). Higher scores indicate a greater sense of inadequacy, self‐hate, or self‐reassurance. For this study, item 6 was replaced with “I do not like being myself” due to the mention of self‐harm.	.82 (IS) .89 (HS) .82 (RS)	.59 (IS) .88 (HS) .76 (RS)
Acceptability	Interest/engagement (immediate postintervention)	4 rating scales (1 to 5) of understanding, interest, topic importance, likelihood of continued use 1 item (yes/no) on whether they would recommend to a friend 4 short‐answer questions: *“what were the top 3 things you learned from taking part?*,*” “What did you like MOST about the lesson?*,*” “What did you like LEAST about the lesson?*,*” “Do you have any other comments?”*	—	—
	Engagement in practice exercises and use of techniques.	2 item (yes/no) assessing use of techniques and engagement in take‐home exercises, with short‐answer to offer reason why/why not 1 short‐answer question providing opportunity for any further comments for improving resources	—	—

## 4 RESULTS

### 4.1 Development

Overall, staff and student feedback was positive. Key modifications included removing gender references from examples to ensure inclusivity, modifying examples to increase age‐appropriateness, and including a topic importance statement. The necessity of a topic importance statement arose in the male focus groups. A common theme among the boys was that they did not find the content relevant but they understood the inherent importance of the topic. The statement in the video highlighted how the lesson can help the students not only support themselves but support others around them, to help foster engagement (see Table S2 for further detail).

### 4.2 Implementation

#### 4.2.1 Feasibility

Regarding uptake, dual parental and student consent was obtained for 128 of a possible 286 students (44.8%). Ten provided active non‐consent, 148 (51.7%) did not return the parental consent form, and 27 did not provide assent or start the survey on the day. Acceptable retention was set at 80% (e.g., Bluth et al., 2016). Of the 101 students who completed the baseline assessment, 79 (78.2%) completed the postintervention assessment, and 85 (84.2%) completed the 1‐week follow‐up, demonstrating reasonable retention (Johnson et al., 2017). Missing data was minimal across outcomes (4 participants at baseline, 11 at immediate postintervention, and none missing at 1‐week follow‐up). Participants were classed as having missing data if they had one or more missing data points within their questionnaire responses. Participants with missing data on trait outcome measures were omitted from trait outcome analysis, while participants with missing data on state outcome measures were omitted from state outcome analysis. All measures showed good internal consistency, except the inadequate‐self subscale of the FSCRS‐SF was poorer among boys (*α* = .59). Despite a technical error in video presentation which can be easily addressed (a default age restriction setting was unknowingly applied), staff reported no difficulties implementing the lesson.

#### 4.2.2 Acceptability

##### Immediate postintervention

Means and standard deviations for quantitative acceptability ratings are found in Table 2. Understanding and topic importance was high; the self‐compassion intervention demonstrated higher acceptability scores for both genders across all outcomes. For students receiving self‐compassion, 100% of girls reported they would recommend to a friend compared with 58.8% of boys. A more even endorsement was observed among boys (66.7%) and girls (64.0%) receiving cognitive‐dissonance.

**TABLE 2:** Programme acceptability and within‐group changes across state and trait outcomes, by gender and intervention condition

	Girls				Boys			
Variable	SC (*n* = 24)	CD (*n =* 25)	*t* (*p*)	*d*	SC (*n* = 16)	CD (*n* = 12)	*t* (*p*)	*d*
	Mean (*SD*)	Mean (*SD*)			Mean (*SD*)	Mean (*SD*)		
Acceptability				
Understanding	4.29 (0.62)	3.92 (0.75)	1.73 (.090)	0.49	4.13 (0.89)	3.83 (0.94)	0.83 (.41)	0.32
Interest	3.96 (0.75)	2.76 (0.97)	4.85 (<.001)	1.38	3.44 (1.32)	2.33 (1.23)	2.28 (.03)	0.86
Topic importance	4.63 (0.58)	4.00 (1.00)	2.69 (.010)	0.76	4.06 (0.93)	3.42 (1.08)	1.66 (.11)	0.65
Likelihood of continued use	3.63 (0.77)	2.72 (1.06)	3.43 (.001)	0.97	3.50 (1.37)	2.75 (1.22)	1.53 (.14)	0.76

	Δ (*SD*)^a^	Δ (*SD*)^a^	SC *d*	CD *d*	Δ (*SD*)^a^	Δ (*SD*)^a^	SC *d*	CD *d*
	(*n* = 24)	(*n* = 23)			(*n* = 14)	(*n* = 7)		
State outcomes								
Weight satisfaction	8.83 (21.64)	−0.30 (25.30)	0.41	0.01	−1.71 (12.85)	2.00 (21.76)	0.13	0.09
Weight distress	−8.13 (23.24)	−7.26 (21.67)	0.35	0.34	−4.50 (23.96)	−3.86 (8.24)	0.19	0.47
Appearance satisfaction	4.46 (15.21)	3.57 (20.96)	0.29	0.17	−3.21 (16.98)	3.43 (10.45)	0.19	0.33
Appearance distress	−6.50 (16.65)	−7.74 (26.60)	0.39	0.29	−2.36 (16.10)	−5.71 (9.74)	0.15	0.59
Positive mood	12.00 (15.37)	−2.57 (16.91)	0.78	0.15	1.36 (19.77)	−13.14 (30.45)	0.07	0.43
Ideal internalization^b^	−14.83 (18.00)	−3.74 (18.54)	0.82	0.20	—	—	—	—
Media pressure	−13.96 (20.79)	−1.04 (19.86)	0.67	0.05	−4.86 (5.83)	−5.00 (8.37)	0.83	0.60
Media pressure distress	−13.50 (20.11)	−0.78 (14.45)	0.67	0.05	−4.00 (7.03)	−5.71 (7.46)	0.57	0.15

	Δ (SD)^c^	Δ (SD)^c^			Δ (SD)^c^	Δ (SD)^c^		
	(*n* = 23)	(*n* = 22)			(*n* = 24)	(*n* = 15)		
Trait outcomes								
Weight and shape concern	−0.30 (0.78)	−0.23 (0.76)	0.38	0.31	0.15 (0.84)	0.54 (1.48)	0.17	0.37
Thin/low body fat internalization	−0.23 (0.67)	−0.18 (0.50)	0.33	0.36	0.10 (0.68)	0.15 (0.92)	0.15	0.16
Athletic/muscular internalization	−0.24 (0.66)	−0.10 (0.52)	0.37	0.19	0.03 (Δ0.46)	−0.20 (0.74)	0.06	0.27
Media pressure	0.75 (1.43)	0.43 (1.21)	0.52	0.36	0.31 (1.18)	0.60 (1.35)	0.26	0.45
Body Appreciation	−0.00 (0.39)	0.16 (0.32)	0.00	0.49	0.05 (0.58)	−0.34 (0.93)	0.09	0.37
Positive affect	0.09 (0.65)	−0.04 (0.59)	0.13	0.06	−0.18 (0.83)	−0.20 (0.96)	0.21	0.21
Negative affect	−0.05 (0.73)	0.37 (0.79)	0.07	0.47	0.29 (0.70)	0.35 (0.75)	0.35	0.47
Self‐compassion	0.20 (0.79)	−0.02 (0.55)	0.25	0.04	0.74 (0.70)	−0.17 (0.60)	1.05	0.28
Inadequate self	−0.26 (0.43)	−0.05 (0.64)	0.60	0.09	0.13 (1.09)	0.65 (0.88)	0.12	0.75
Hated self	0.01 (0.52)	0.25 (0.53)	0.02	0.47	0.38 (1.06)	0.58 (1.06)	0.35	0.55
Reassured self	−0.13 (0.64)	−0.00 (0.60)	0.20	0.00	−0.17 (0.63)	−0.27 (0.68)	0.26	0.39

*Note*: One student identifying as non‐binary was not able to be included in gender analysis.

Abbreviations: CD, cognitive dissonance; *d*, Cohen's *d* effect size (0.2 = small, 0.5 = medium, 0.8 = large); SC, self‐compassion.

^a^ **Δ** represents change to immediate postintervention (immediate postintervention – baseline).

^b^ State ideal internalization change was not analyzed in boys due to a wording error mentioning female body ideals.

^c^ **Δ** represents change to 1‐week follow‐up (1‐week follow‐up – baseline).

Students in both conditions reflected aspects they had learnt that were congruent with intervention content themes. Most liked elements were also similar, including content themes, relevance, video format, use of “real‐world” examples and the ability to complete in‐class worksheets at their own pace. Least liked elements included the length of the survey, length of lesson and time given for the in‐class worksheet, and desire for more engaging video presentation. Content not being relevant was more commonly raised among boys, consistent with focus group feedback. Nevertheless, many boys did engage and provided positive feedback, and reflected importance of the topic for others if not themselves.

##### One‐week follow‐up

For self‐compassion, substantially more girls (60.9%) than boys (16.7%) reported using the techniques during the week. Conversely, more boys (26.7%) used the cognitive‐dissonance techniques than girls (4.5%). Reasons for using the techniques mostly reflected feeling they were or would be helpful; common reasons for not engaging were lack of interest, relevance, time, or forgetting. Regarding the take‐home worksheets, 43.5% of girls and 20.8% of boys in the self‐compassion condition and 22.7% of girls and 20% of boys in the cognitive‐dissonance condition reported completion. The most common responses for non‐engagement again cited lack of interest, relevance, time or forgetting. Some students stated they did not receive the take‐home sheets.

#### 4.2.3 Within‐group changes in outcomes

Mean change from baseline to postintervention and 1‐week follow‐up are presented in Table 2. The majority of state outcomes showed a favorable direction of change, particularly among girls receiving self‐compassion and boys receiving cognitive‐dissonance. Unexpectedly, there was a decrease in positive mood among boys receiving cognitive‐dissonance, which was commensurate with positive changes on other outcomes. A mixed pattern of changes in trait outcomes was observed across condition and gender, although reflects some promising indications of support for intervention targets (e.g., increases in self‐compassion for self‐compassion training, reduction in athletic‐ideal internalization in boys and thin‐ and athletic‐ideal internalization in girls for the cognitive‐dissonance training).

## 5 DISCUSSION

The current study investigated the acceptability and feasibility of two microinterventions focused on responding to appearance‐ideal media using self‐compassion and cognitive‐dissonance techniques, respectively, among adolescents in a school setting.

Feasibility of intervention implementation and research methods relevant for a further trial was indicated based on assessment retention, measure completion, and minimal reported classroom difficulties. Despite consent approaching just 45%, the number of students providing active non‐consent was low (3.5%). More time should be allocated for recruitment, as well as providing education to parents about the benefits of measuring outcomes of wellbeing initiatives. Retention rates were favorable and generally consistent with other school‐based universal prevention programs targeting adolescents (e.g., Johnson et al., 2017); reasons for missing data were not collected and could be included in future to further understand feasibility and biases.

Student acceptability was promising, with over 70% of participants stating they would recommend the lesson, consistent with single and multisession universal school‐based programs (e.g., 70%–83%; Garbett et al., 2022). The self‐compassion intervention demonstrated greater acceptability, particularly among girls, consistent with research in adults (e.g., Toole et al., 2021). Self‐compassion interventions may appeal more to females; they do not attempt to explicitly target appearance ideals as in dissonance‐based interventions, which may be tightly held and therefore uncomfortable to challenge, but instead, aim to provide tools to cope with sociocultural pressures which may feel less confronting (Toole et al., 2021).

In their respective conditions, males demonstrated consistently lower acceptability. There is limited research investigating adolescent boys' acceptability of ED prevention programs generally. However, masculine norms, which encourage men to discount their emotions, often prevent males from engaging in self‐help interventions/activities and may have contributed to lower acceptability (Heath et al., 2017). Self‐compassion techniques may be especially challenging as the construct appears incongruent with masculine norms, which often promote self‐criticism (Reilly et al., 2014).

Despite lower acceptability, removing males from prevention efforts has the potential to do harm, by promoting the concept that body image is a female‐only issue, and further perpetuating toxic masculine norms. Additionally, separating students into “males” and “females” to receive a separate intervention goes against our continuously evolving understanding of gender as non‐binary. For example, drive for muscularity is often described in male ED research, however, recent research suggests muscularity concerns are becoming a core component of the female ideal due to media influences (Cramblitt & Pritchard, 2013; Girard et al., 2018). Given these factors, combined with the time‐limited nature of microinterventions, and the practical difficulty of separating genders, it is recommended that implementation should continue to be delivered universally (see Table 3). This issue should nonetheless be revisited within larger scale evaluation, including opportunities for some level of personalized content matching as also suggested in Atkinson and Diedrichs (2021).

**TABLE 3:** Recommendations for future use of interventions based on staff and student feedback

1	Present the video interventions via main classroom screen rather than individual devices (to reduce opportunity for distraction)
2	Target age to remain the same (15–17 years old)
3	Continued participation by all genders. Although males generally reported less interest in the lessons, many males did engage and find the lessons useful. Further consultation with male‐identifying students will help to maximize relevance.
4	Further adaptation of the materials based on student feedback and recommendations (mainly including shortening the length of the videos further and more varied presentation style) *Students should be involved in co‐creation of videos (scripts and delivery decisions) for future implementation, and potentially be a part of the filming*.
5	Consider additional options for delivery as suggested by students, e.g., involving group discussion during and after the video presentation, after sharing supportive discussion tips with classroom teachers
6	Re‐consider whether take‐home sheets are implemented or how they may be re‐framed or incorporated into a further short lesson, based on further consultation with teachers and students
7	Ultimately, a stronger evaluation of the effectiveness of the interventions should be undertaken with inclusion of a control comparison group.

Despite promising lesson acceptability, completion of the take‐home sheet was poor for all groups. Poor acceptability of homework in school prevention programs is common (e.g., Atkinson & Wade, 2015) and in the current study was unsurprising due to the concerns regarding completion raised in the focus groups (see Table S2). However, due to the brevity of the microinterventions, ongoing reflection and practice is likely important to promote a longer‐lasting impact. Future studies should consider how best to facilitate this, for example, through making homework compulsory, using gamification to improve engagement, or completing the sheets during class time (see Table 3).

Within‐group differences between baseline and follow‐up across risk factors for EDs generally showed a favorable direction of change, particularly on state measures which is in keeping with the targeted nature of microinterventions to provide immediate impacts (Elefant et al., 2017). The most consistent changes were seen for sociocultural risk factors, consistent with Atkinson and Diedrichs (2021) and aligned with the intervention focus on responding to appearance pressures in the media. Positive mood decreased among participants engaging in cognitive‐dissonance induction, which may reflect the more challenging nature of counter‐attitudinal content. Despite more mixed findings, some promising trait changes emerged, providing preliminary support for intervention mechanisms for both self‐compassion and dissonance‐induction. Trait media pressure increased at 1‐week follow‐up for all groups despite state media pressure demonstrating favorable changes. Diedrichs et al. (2015) found a similar result with elevated perceived sociocultural pressures following a single‐session body‐image lesson. This effect was not maintained at follow‐up and is potentially due to raised *awareness* of pressures due to the nature of intervention content and assessment. Further evaluation is nevertheless essential to confirm no harm for participants where less favorable changes were observed.

### 5.1 Limitations and future research

The current study provides encouraging support for the feasibility and acceptability of two video‐based microinterventions for use among mid‐adolescents in a school setting, however, some caution should be taken. Due to the preliminary nature (including small sample and lack of control comparison), no statistical inferences should be made concerning outcome change. The within‐group changes discussed in the current study should be regarded as exploratory. These changes were analyzed to help determine whether proceeding with developing the interventions is advisable and to guide measure selection for a full‐scale trial within this setting. Further, the study recruited a single private co‐educational school with good access to well‐being services, limiting generalizability. Finally, students who participated in the focus groups were not excluded from data analysis. Despite the differing purpose, delivery format, and subsequent intervention modifications, these students nevertheless were exposed to the intervention twice which may have influenced results.

Future research should include further modifications, based on current findings and additional qualitative methods (e.g., think‐aloud interviews; Yardley et al., 2015) to increase acceptability, especially among males (Table 3 outlines more detailed recommendations). This should also explore the best way to facilitate the take‐home exercises, as noted above. Further evaluation should increase representation across school characteristics and include a control and/or active comparison to robustly assess efficacy.

Overall, the study findings support the further development and efficacy trial of self‐compassion and dissonance‐based microinterventions in secondary schools, to provide focused, accessible tools for staff and students in time‐poor educational settings.

## AUTHOR CONTRIBUTIONS

**Mhairi Kristoffersen:** Data curation; formal analysis; methodology; writing – original draft; writing – review and editing. **Catherine Johnson:** Conceptualization; methodology; supervision; writing – review and editing. **Melissa J Atkinson:** Conceptualization; methodology; supervision; writing – review and editing.

## CONFLICT OF INTEREST

The authors have no conflicts of interest to declare.

## Supporting information


**Table S1** Outline of intervention content.
**Table S2** Intervention modifications made during the development phase based on school staff and student consultation, and in line with guiding principles.

## Data Availability

Data available on request due to privacy/ethical restrictions.
