# Supplementary material for: Feasibility and acceptability of video‐based microinterventions for eating disorder prevention among adolescents in secondary schools
Source: Int J Eat Disord. 2022 Jul 19;55(11):1496–505. doi: 10.1002/eat.23781 (PMC9796454; doi:10.1002/eat.23781)
Supplement: Supplementary file 1 — Table S1 Outline of intervention content. Table S2 Intervention modifications made during the development phase based on school staff and student consultation, and in line with guiding principles. [file EAT-55-1496-s001.docx]

Supplementary Materials

**Table S1**

*Outline of Intervention Content*

| **Cognitive dissonance (adapted from Atkinson & Diedrichs, 2021; Stice & Presnell, 2007)** |
| --- |
| 1. Introduction into the role the media plays in promoting unrealistic body ideals and the importance of learning strategies to respond to these effectively 2. Students guided through thinking about what unrealistic ideals are presented in the media and how these ideals can impact young people 3. Students then directed to the first exercise on the in-class worksheet asking them to consider the ‘costs’ of pursuing appearance ideals, including emotional, health, school/extracurricular, financial and societal costs. 4. Focus then on how to challenge the appearance ideals that the media promotes, including examples 5. Students were then directed to the in-class work sheet which provided example scenarios in which participants were to create and write down a response to *(e.g. You are a friend are scrolling through Instagram. Your friend says that they wished they looked like the influencers that appear on your feed. What could you say to your friend to convince them that it’s not worth pursuing this ‘ideal’ body?)* 6. Focus then on the importance of practicing the techniques used in the video and challenging appearance pressures. Participants were asked to practice over the coming week. 7. A take-home worksheet was provided. The worksheet included a summary of what was covered in the video and instructions for practice exercises. |
| **Self-compassion (adapted from Atkinson & Diedrichs, 2021; Atkinson & Wade, 2015; Gilbert, 2010)** |
| 1. Introduction into the role the media plays in promoting unrealistic body ideals and the importance of learning strategies to respond to these effectively 2. Introduction to the concept of self-compassion and that it can help manage negative thoughts and feelings and improve well-being (noting that even if students feel unaffected the lesson can still help them help others around them) 3. Highlighting cons of self-criticism utilising an example comparing approaches of two different sports coaches (Coach Critical versus Coach Compassion). Students were directed to the first exercise on their in-class worksheet asking follow-up questions (e.g., what impact would each coach have on your thoughts about yourself?) 4. Discussion around costs of being self-critical, with examples (e.g., setting appearance goals that are unhealthy). Students were then directed to the second exercise on the in-class worksheet asking them to note down the costs of being self-critical about their body shape/appearance 5. Rationale presented for how self-compassion provides a different way of responding to negative experiences and how it can be helpful in some situations, with examples. Students were directed to the worksheet to practice developing self-compassion via generating self-compassionate responses to scenarios provided (e.g., comparing to people in Tik Tok videos) 6. Encouraged on the importance of practising the techniques used in the video and being self-compassionate. Participants were asked to practice over the coming week. 7. A take-home worksheet was provided. The worksheet included a summary of what was covered in the video and instructions for practice exercises |

**Table S2**

*Intervention Modifications Made During the Development Phase Based on School Staff and Student Consultation, and in line with Guiding Principles*

| **Staff Feedback** | **Modification Made/Reason for no modification** | **Relevant Guiding Principles** |
| --- | --- | --- |
| *General* |  |  |
| Individuals delivering the intervention via video should be young but older than the students (i.e., 18-21) to make content more relatable and engage students. | The author (22) narrated the video alongside another presenter (male, 22) to make the video relatable and diverse. | Ensure content is relevant and accepted by mid-adolescents. |
| *Cognitive Dissonance* |  |  |
| Tasks are quite challenging and confronting. With students that struggle with critical analysis, their responses may be brief. | Extra examples of possible answers were included on the worksheet and were also added to the video script. | Provide easy exercises to help promote understanding during the intervention. |
| For the take-home exercise, ensure the students know that it is a hypothetical letter and will not be sent, which may prevent some students from completing it. | Change the description of the task, mentioning that is it hypothetical and that the students will not be asked to send the letter. | Ensure content is accepted by mid-adolescents. |
| *Self-compassion* |  |  |
| Remove names (Smith etc.) and genders from the coach example to make it more diverse and reduce the chance of upset if names match students. | Remove names and utilise Coach Compassion and Coach Critic. Utilise gender-neutral pronouns (they/them) to aid acceptability and diversity and reduce any potential bias. | Provide content that is not exclusive to one gender (to make intervention accessible to all genders). |
| **Student Feedback** | **Modification Made/Reason for no modification** | **Guiding Principles** |
| *General* |  |  |
| Content and the general topic may not be as relatable to males. It may be helpful to mention that even if it doesn't apply to you, it may help you support people that it does affect to make sure everyone is included making it more likely that everyone pay's attention. | After the introduction, it was explained that even if the students didn't feel like the lesson was relevant to them, it could still help them support others such as their friends and family to whom it is relevant. This is to try and engage those who may feel like it isn't relevant to them. | Ensure content is relevant and accepted by mid-adolescents and make the intervention accessible to all genders. |
| It would be useful to work in groups to share ideas of what people came up with in case people struggle or miss things. | Due to the design of the interventions being self-led and easy to implement in schools, this change was not made to make it as feasible and straightforward as possible. This is something to consider, however, when analysing results and for future considerations. | N/A |
| People are unlikely to do the take-home sheet. Doing it in class would mean more people would do it. | Out with the scope of the study. Intervention is designed to be student-led and easy for schools to implement (within one lesson)—something to consider in future. | N/A |
| It would be helpful to do a discussion at the end to share ideas. | Despite this suggestion, many students also believed the effectiveness of this would be too dependent on the students in the class and the class teacher. Therefore, to keep the intervention brief and student-led, this modification was not implemented. | N/A |
| If individuals, do it on their individual computers, they may get distracted and start playing games or doing something else. | This modification was initially implemented. However, due to the numbers of non-consenting students in classes and COVID-19, showing the video to the whole class was not feasible for the current study. | N/A |
| *Cognitive Dissonance* |  |  |
| The example in the third section concerning what an individual may say to themselves is long and unrealistic. | Example shortened to one sentence instead of two to translate better to real-life situations and make it more representative of what someone may say to themselves. | Utilise examples relevant to mid-adolescents (Ensure content is relevant and accepted by adolescents). |
| *Self-Compassion* |  |  |
| The bike analogy used doesn't make sense and wasn't relatable. | The analogy was modified to be directed towards academic tests (as suggested by participants) to make it more relatable. | Utilise examples that are relevant to mid-adolescents |
| It's not clear why the first task was related to a sibling or a friend and not themselves. It would be more valuable if it was related to themselves. | The task was changed to be related to the students themselves, rather than a sibling or friend to enhance acceptability—something to consider further based on additional student feedback post-intervention. | Utilise examples that are relevant to mid-adolescents (Ensure content is relevant and accepted by adolescents) |
